# Concurrent trastuzumab deruxtecan-induced interstitial lung disease and COVID-19 in the treatment of advanced breast cancer

**DOI:** 10.1093/omcr/omad135

**Published:** 2023-12-19

**Authors:** Naoaki Yasuda, Satoshi Ikeo, Akihiko Sokai, Yuki Sakai, Yasuyuki Hayashi, Sae Kitano, Naoko Itoi, Tecchuu Lee, Toshiyuki Iwata, Takashi Nishimura

**Affiliations:** Department of Respiratory Medicine, Kyoto Katsura Hospital, Kyoto, Japan; Department of Respiratory Medicine, Kyoto Katsura Hospital, Kyoto, Japan; Department of Respiratory Medicine, Kyoto Katsura Hospital, Kyoto, Japan; Department of Respiratory Medicine, Kyoto Katsura Hospital, Kyoto, Japan; Department of Respiratory Medicine, Kyoto Katsura Hospital, Kyoto, Japan; Department of Breast Surgery, Japanese Red Cross Kyoto Daiichi Hospital, Kyoto, Japan; Department of Surgery, Division of Endocrine and Breast Surgery, Kyoto Prefectural University of Medicine, Kyoto, Japan; Department of Breast Surgery, Japanese Red Cross Kyoto Daiichi Hospital, Kyoto, Japan; Department of Breast Surgery, Japanese Red Cross Kyoto Daiichi Hospital, Kyoto, Japan; Department of Respiratory Medicine, Kyoto Katsura Hospital, Kyoto, Japan; Department of Respiratory Medicine, Kyoto Katsura Hospital, Kyoto, Japan

**Keywords:** trastuzumab deruxtecan, antibody-drug conjugate, drug-induced interstitial lung disease, COVID-19

## Abstract

Patients with cancer are at an increased risk of developing coronavirus disease 2019 (COVID-19) infection. Trastuzumab deruxtecan (T-DXd) is an antibody-drug conjugate (ADC) against epidermal growth factor receptor 2 (HER2)-positive cancer, known to cause drug-induced interstitial lung disease (DILD), including drug-induced pneumonitis. A 60-year-old woman with breast cancer developed a fever during treatment with T-DXd and was diagnosed with COVID-19. The fever persisted for approximately 3 weeks, and chest computed tomography showed multiple consolidations with bilateral peripheral predominance. Since the clinical course was atypical for COVID-19 due to the long duration of the fever and the CT pattern was frequently seen in T-DXd-induced ILD, the patient was diagnosed with T-DXd-induced ILD, following which, prednisolone was started, leading to improvement in the symptoms and fading of shadows. Even in patients suspected of COVID-19 pneumonia, physicians should consider the possibility of DILD, particularly in patients undergoing cancer treatment.

## INTRODUCTION

Patients with cancer are at an increased risk of developing coronavirus disease 2019 (COVID-19) infection [1]. Although nearly 4 years have passed since the outbreak, the COVID-19 pandemic has not yet shown any signs of resolution. The Wuhan strain was the predominant strain in 2020, and the typical clinical course was progression to respiratory failure within approximately 8 days, followed by acute respiratory distress syndrome and multiorgan failure within approximately 16 days after the initial onset of symptoms [2]. Non-specific viral symptoms, such as fever, cough, and runny nose, were also usually present [2]. Trastuzumab deruxtecan (T-DXd), an antibody-drug conjugate (ADC) against human epidermal growth factor receptor 2 (HER2), is used for the treatment of advanced HER2-positive breast cancers and is known to cause drug-induced interstitial lung disease (DILD), including drug-induced pneumonitis [3, 4]. DILD causes fever as well as respiratory symptoms, such as dyspnoea and cough [5]. Herein, we describe a case of COVID-19 diagnosed during breast cancer treatment, complicated by DILD due to T-DXd.

## CASE PRESENTATION

**Figure 1:**
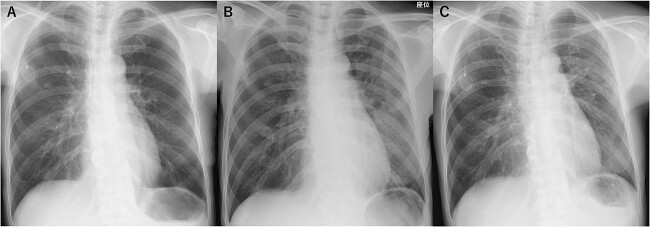
A series of chest radiographs. (**A**) On admission—no abnormalities other than a known lung metastasis in the right middle lung region. (**B**) Day 20—infiltrative shadow in the left middle lung region. (**C**) Day 34—shrinkage of infiltrative shadow.

**Figure 2:**
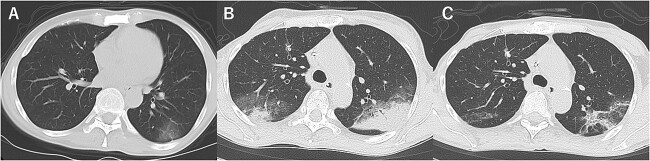
A series of chest computed tomography images. (**A**) On admission—ground-glass opacity in the left lower lung lobe. (**B**) Day 20—Sub-pleural, multiple consolidations in both upper lungs. (**C**) Day 34—fading consolidation.

A 60-year-old woman with no smoking history presented with a fever. She had no significant medical history apart from a lack of vaccination against COVID-19 and recurrent breast cancer for which she was undergoing treatment with T-Dxd since past 5 months, and the T-DxD administration was repeated every 3 weeks without significant adverse events. She also did not have renal failure. Her husband, who lived with her, had already been diagnosed with COVID-19. No significant symptoms were observed other than the fever. No hypoxemia was observed, and the patient had normal breath sounds. Blood tests revealed a white blood cell count of 2810/μl and C-reactive protein level of 0.13 mg/dl. Chest radiography showed no abnormalities other than a known lung metastasis in the right middle lung region (Fig. 1A); however, chest computed tomography (CT) showed a ground-glass opacity in the left lower lung lobe (Fig. 2A). Polymerase chain reaction (PCR) on nasopharyngeal swabs for severe acute respiratory syndrome coronavirus 2 (SARS-CoV-2) confirmed the diagnosis of COVID-19. No respiratory failure was observed; therefore, the patient was followed up without COVID-19 treatment. However, because of prolonged fever, SARS-CoV-2 PCR tests were performed on the 18th and 19th days of illness onset and were negative on both days. On day 20, chest radiography showed an infiltrative shadow in the left middle lung region (Fig. 1B), and chest CT revealed multiple consolidations with peripheral predominance in both lungs (Fig. 2B). There was no symptom suggestive of bacterial pneumonia, such as yellow sputum, and bacterial culture could not be performed. As the patient had been diagnosed with COVID-19 infection, we decided against performing a bronchoscopy to avoid spreading the COVID-19 infection. Considering the patient’s long duration of fever and CT pattern, which is frequently observed in DILD caused by T-DXd, she was diagnosed with T-DXd-induced ILD grade 2 per the Common Terminology Criteria for Adverse Events (CTCAE) version 5.0 scale, and 1 mg/kg prednisolone (45 mg/body weight) was administered on day 21. After starting prednisolone, the fever resolved on day 32, and chest radiography and CT on day 34 showed shrinkage of the infiltrative shadows (Figs 1C and 2C). There were no complications with prednisolone treatment following which prednisolone was tapered to 25 mg/body weight and the patient was discharged on day 42 after onset.

## DISCUSSION

This is a case of coexisting DILD caused by T-DXd and COVID-19. Despite the absence of respiratory failure, the patient in this case had a fever for almost 20 days, which was significantly longer than the average for COVID-19 patients. The median duration of fever in COVID-19 patients who were not admitted to the ICU was 10 days (95% confidence interval: 8–11 days) [6]. Although blood tests can hardly differentiate COVID-19 infection from other diseases, this patient’s clinical course was unusual for COVID-19 and led us to consider causes other than COVID-19 for the fever. We suspected DILD by T-DXd because of her treatment history of breast cancer. Since the husband, who lived with her, also had COVID-19 at the same time, a false positive result of the COVID-19 PCR test was considered unlikely.

An important point to ponder in this case is the high incidence of DILD with T-DXd therapy. Tamura et al. reported that among 115 patients with HER2-positive advanced-stage breast cancer treated with T-DXd, 20 (17.4%) developed DILD, of whom six developed organizing pneumonia, and the median time from the start of treatment to the onset of DILD was 8.3 months [3]. The clinical course of this case was also similar to that of this study; approximately 5 months had passed after T-DXd administration, and the imaging pattern was that of organizing pneumonia, and this case is consistent with DILD due to T-DXd. However, Chest CT features of COVID-19 are ground glass opacities with and without consolidation, multiple lesions, bilateral involvement, posterior part and/or lower lobe predilection, and peripheral and/or subpleural distribution [7], similar to the CT images of organizing pneumonia. In this case, the CT pattern of the patient was indistinguishable from that of COVID-19 pneumonia. In addition, after experiencing this case, a series of case reports of organizing pneumonia post-COVID-19 were published [8–10]. These reports indicate that it is extremely difficult to distinguish between the two aetiologies—T-DXd-induced ILD and COVID-19-related pneumonia—with CT images because both have the same pattern of organizing pneumonia. However, of the 1272 COVID-19 reported patients, only 35 (2.8%) had a CT imaging pattern of organizing pneumonia [8], which is much rarer than DILD due to T-DXd; therefore, in light of this, we considered that this case was most likely due to organizing pneumonia related to DILD with T-DXd.

In 2022, a multicenter, international phase 2 study of T-DXd in patients with HER2-positive non-small cell lung cancer (NSCLC) refractory to standard therapy was reported [11]. The median overall survival was 17.8 months, 24 of 91 patients (26.3%) developed DILD, and 2 patients died. Steroid administration is considered for grade 1 ILD and is recommended for grade 2 or higher ILD. In this case, the patient had developed grade 2 DILD per the CTCAE version 5.0. T-DXd was withdrawn, and steroid therapy was initiated rather than reducing the dose of T-Dxd, according to a previous study’s protocol [3]. Interestingly, the incidence of DILD with trastuzumab emtansine (T-DM1), another antibody-drug conjugate (ADC) against HER2, was lower (10/884, 1.1%) [12]. Although both ADCs contain trastuzumab, T-DM1 is a drug conjugated with a microtubule polymerization inhibitor, whereas T-DXd is conjugated with a topoisomerase I inhibitor. Both interventions may have different mechanisms against cancer, leading to the difference in the frequency of DILD; however, the exact reason is unknown and needs further investigation.

This case highlights the need to be aware of conflating symptoms to COVID-19 alone; especially even when COVID-19 is diagnosed, other differentials should be considered, despite the ongoing pandemic. Additionally, as mentioned, T-DXd is expected to be used in the future treatment of various cancers; therefore, physicians should be fully aware of the DILD risks of ADCs against HER2. Important aspects of management include confirming the diagnosis by exclusion and appropriate disease management, including proper steroid use. In addition, investigating the reason for the differing frequency of DILD observed between T-DXd and T-DM1, which are similar drugs, may lead to a better understanding of the molecular biological mechanisms underlying DILD.

## Data Availability

The data are available within the article.
